# Step-By-Step Instructions for Retina Recordings with Perforated Multi Electrode Arrays

**DOI:** 10.1371/journal.pone.0106148

**Published:** 2014-08-28

**Authors:** Katja Reinhard, Alexandra Tikidji-Hamburyan, Hartwig Seitter, Saad Idrees, Marion Mutter, Boris Benkner, Thomas A. Münch

**Affiliations:** Werner Reichardt Centre for Integrative Neuroscience and Bernstein Center for Computational Neuroscience, University of Tübingen, Tübingen, Germany; Dalhousie University, Canada

## Abstract

Multi-electrode arrays are a state-of-the-art tool in electrophysiology, also in retina research. The output cells of the retina, the retinal ganglion cells, form a monolayer in many species and are well accessible due to their proximity to the inner retinal surface. This structure has allowed the use of multi-electrode arrays for high-throughput, parallel recordings of retinal responses to presented visual stimuli, and has led to significant new insights into retinal organization and function. However, using conventional arrays where electrodes are embedded into a glass or ceramic plate can be associated with three main problems: (1) low signal-to-noise ratio due to poor contact between electrodes and tissue, especially in the case of strongly curved retinas from small animals, e.g. rodents; (2) insufficient oxygen and nutrient supply to cells located on the bottom of the recording chamber; and (3) displacement of the tissue during recordings. Perforated multi-electrode arrays (pMEAs) have been found to alleviate all three issues in brain slice recordings. Over the last years, we have been using such perforated arrays to study light evoked activity in the retinas of various species including mouse, pig, and human. In this article, we provide detailed step-by-step instructions for the use of perforated MEAs to record visual responses from the retina, including spike recordings from retinal ganglion cells and *in vitro* electroretinograms (ERG). In addition, we provide in-depth technical and methodological troubleshooting information, and show example recordings of good quality as well as examples for the various problems which might be encountered. While our description is based on the specific equipment we use in our own lab, it may also prove useful when establishing retinal MEA recordings with other equipment.

## Introduction

Multi-electrode arrays (MEAs) are a state-of-the-art tool in electrophysiological studies. Such arrays consist of dozens up to thousands of electrodes and allow measurements of many neurons in parallel. Especially in retina research, MEA recordings have proven to be a powerful technique [1]–[5]. The retina consists of many parallel yet interacting neural circuits which extract specific information about the visual input [6]. These circuits culminate at the output neurons of the retina, the retinal ganglion cells. The retina’s layered structure with ganglion cells lying close to the proximal surface makes the retina particularly amenable for MEA recordings. Further, in many common laboratory species, including mouse, the ganglion cells form a monolayer with little or no three dimensional piling of cell bodies. This monolayer is covered only by the relatively thin inner limiting membrane, such that these neurons and the flat recording array can be brought into close proximity.

When performing in-vitro MEA recordings with retina, the retina is extracted from the eye and placed ganglion cell-side down on the electrodes of the MEA. Light stimulation is then applied either from the top or, if the MEA is transparent, through the MEA from the bottom. The photoreceptors capture the light and the visual information is processed by the retinal circuits, eventually leading to spike generation in the ganglion cells. These spikes can be measured as voltage changes by the electrodes of the MEA.

Retinal recordings with standard MEAs suffer from three main problems: (1) poor signal-to-noise ratio due to insufficient contact (physical proximity) between the tissue and the MEA (this problem is particularly pronounced when recording from small retinas, e.g. mouse, due to the strong curvature of the retina, and when recording from retinas with a thick inner limiting membrane, e.g. human), (2) insufficient oxygenation and nutrient supply to the ganglion cells lying on the bottom of the recording chamber, and (3) movement of the retina due to insufficient fixation of the tissue on the array. Poor electrode contact and fixation of the tissue are usually dealt with by using some sort of “stamp”, pushing the tissue against the MEA. This has obvious disadvantages, as one needs to find a fine balance between sufficiently holding the tissue in place on one hand, and not damaging the tissue by applying too much pressure on the other hand.

**Figure 1:**
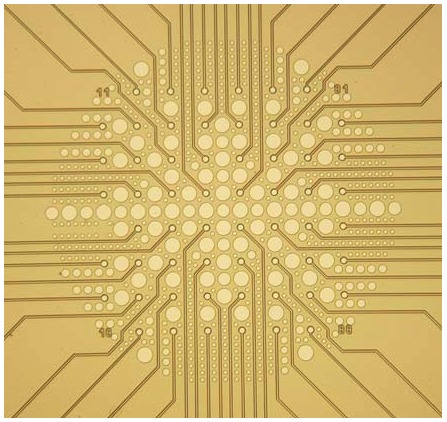
Layout of the 60-electrode pMEA. The electrodes are arranged in an 8×8 array with 200 µm electrode distance. Perforations of various size are visible in-between electrodes (source: 60pMEA200/30iR data sheet by Multi Channel Systems).

In our laboratory, we have implemented retinal recordings with perforated MEAs (pMEAs, from Multi Channel Systems MCS GmbH, Reutlingen, Germany). We found that pMEAs can alleviate all three issues encountered with standard MEAs. In pMEAs, the electrodes are not embedded into a ceramic or glass carrier, but instead in a fine membrane which also contains small holes of different sizes in-between the electrodes (Fig. 1). A slight vacuum can be applied through this perforation; this vacuum gently pulls the tissue towards the electrodes. This procedure enhances the contact between the tissue and the electrodes, and therefore increases the signal-to-noise ratio and decreases tissue movement during the experiment [7]. Additionally, it has been shown with brain slices that with pMEAs, more fresh solution reaches the bottom cell layers either through the tissue or through the small space between tissue and electrodes. Oxygenation of the bottom cell layer (i.e. ganglion cells in the case of retina) is thereby greatly enhanced when using pMEAs [8].

Several adjustments are necessary compared to the procedures applicable to brain slices [9]–[13]. The main reason is that the retina is relatively thin and fragile compared to brain slices, so that the vacuum needs to be very carefully controlled to prevent tearing of the retinal tissue. In this article we give a detailed description of our recording setup for perforated MEAs and step-by-step instructions for two different applications (spike recordings and *in vitro* electroretinogram recordings). We show example data demonstrating recording stability in long-term experiments, and provide an overview of the outcome which can be expected from such measurements. In addition, we discuss possible technical issues and provide troubleshooting suggestions.

## Material

### Perforated MEAs (60pMEA200/30iR by Multi Channel Systems MCS GmbH)

The 60pMEA200/30iR is a pMEA with 60 Titanium nitride electrodes. The electrodes are arranged in an 8×8 layout with 200 µm electrode distance and 30 µm electrode diameter. Electrodes are embedded in a perforated polyimide foil which allows perfusion and application of negative pressure to the retina (Fig. 1, further details can be found in the pMEA data sheet [14]). pMEAs are transparent and can therefore be used in upright and inverted setups. In this article we describe our experiments performed with a 60-electrode pMEA with glass ring and the MEA1060 amplifier. However, recordings with other pMEA systems should require only slight adaptations.

### Tissue

In previous studies we have used pMEAs in many experiments with retinas of several species. In the section “anticipated results” we discuss the quality of data to expect from retinas of various mouse strains, domestic pig retinas (sacrificed during independent studies at the Department of Experimental Surgery, Tübingen), Göttingen minipig retinas (Department of Urology) and human retinas (donated by patients of the University Eye Hospital in Tübingen). All recordings have been performed in the context of scientific studies in our laboratory. All studies were performed in accordance with German and European regulations. Use of human retinal tissue was approved by the Ethics Commission of the University Clinic Tübingen, approval number 531/2011. Written informed consent of the donors was obtained; the consent procedure was part of the Ethics Commission approval. Animal experiments were approved by the Regierungspräsidium Tübingen.

### Setup components

**Figure 2:**
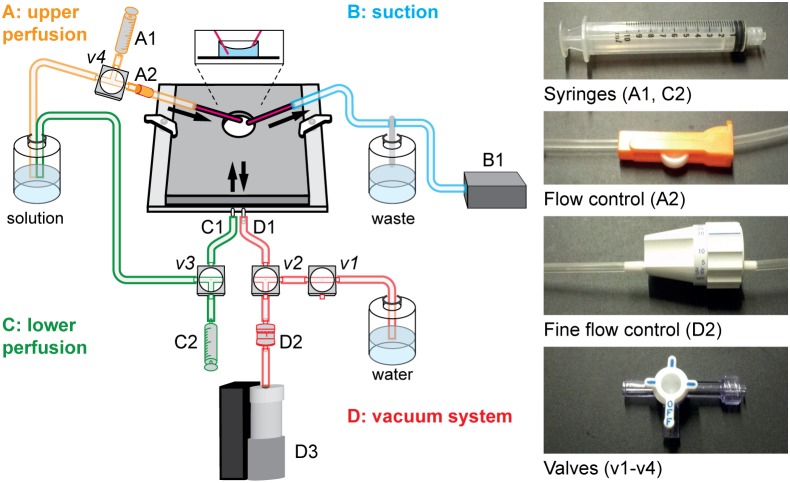
Setup for pMEA recordings. Our MEA setup consists of two perfusion loops. Solution is supplied to the MEA chamber from the top through the upper perfusion (A) and excessive solution is removed by the suction (B). The necessary negative pressure is supplied by the additional perfusion, consisting of the lower perfusion (C) and a vacuum (D). Details are given in the following text and figures.

The setup for pMEA recordings consists of two perfusion loops: An upper loop to supply the tissue with fresh solution (labeled “upper perfusion” and “suction” in Fig. 2), and a lower loop to adjust the proper negative pressure (“lower perfusion” and “vacuum system”). Here, we provide an overview of this dual perfusion system and a detailed list of the components we used to build our setup (excluding light stimulation and data acquisition). Details on how to use the system are described below in the section “experimental procedure”. Except for the constant vacuum pump (D3), the amplifier baseplate that allows vacuum application, and some small components such as tubing, no additional material is needed compared to conventional MEA recordings. All numbers refer to Figure 2.

#### Upper perfusion

The upper perfusion system supplies the retina with fresh solution during the recordings. It can either be gravity driven (like in the scheme in Fig. 2, in which case the tubing can initially be filled with the help of a syringe, A1, v4), or it can be driven with a peristaltic pump. The solution is guided into the MEA chamber through a cannula or a stiff tube. A simple flow regulator (A2) can be used to adjust the speed of the solution flow in the gravity driven configuration. The components used for upper perfusion are listed below.

− Bottle with physiological solution
− 10–20 ml syringe (A1)
− Simple flow regulator (A2, e.g. Infudrop, Fresenius Kabi AG, Bad Homburg, Germany)
− Valve (v4)
− Cannula or similar
− Tubing (inner diameter)
  ▪ 2×∼1.6 mm to connect v4–A2 and A2–MEA
  ▪ 2×∼1.6 mm (or thicker) to connect v4–solution and v4–A1
  ▪ Thinner tubes to connect to cannula (depending on cannula)

− Connectors for attaching the tubing to the other components

#### Suction

To prevent the MEA chamber from overflowing, a suction pump (B1) should be connected via a cannula to the MEA chamber. The solution can either be collected in an extra bottle and discarded after the experiment or, if the upper perfusion is performed with a peristaltic pump, it can be recycled and pumped back into the main solution bottle. The components for suction are listed below.

− Vacuum pump (B1)
− Bottle with gas washing bottle head
− Cannula or similar
− Tubing and connectors, appropriate to fit attachments for waste bottle and pump

#### Lower perfusion

The lower perfusion system is only used before the experiment and can be driven by gravity flow. Its purpose is to fill the MEA chamber with solution without introducing air bubbles into the vacuum system. The lower perfusion is connected to the shorter cannula of the pMEA amplifier baseplate (C1). To get the gravity-driven flow going, the tubing of the lower perfusion system can be filled with the help of a syringe (C2, v3). The components for lower perfusion are listed below.

− 10–20 ml syringe (C2, with screw connection for valve)
− Valve (v3)
− Tubing (inner diameter)
  ▪ 1×0.8 mm to connect v3–C1
  ▪ 1×∼1.6 mm to connect v3–solution

− Connectors at v3

#### Vacuum system

The vacuum system provides negative pressure to pull the retina towards the electrodes. This negative pressure needs, first, to be constant to avoid fluctuations, and second, to be high enough to ensure good tissue-electrode contact, but low enough to not tear the tissue. Constant negative pressure is provided by a Constant Vacuum Pump (CVP, D3, Multi Channel Systems) and is further reduced by an additional fine flow control (D2) between the CVP (D3) and the MEA baseplate. The vacuum system is connected to the right (longer) cannula of the MEA baseplate (D1). The most important step for ensuring reliable negative pressure is the removal of air bubbles: any air bubble in the vacuum system will degrade the stable negative pressure. The additional valves (v1, v2) and the water bottle are needed for filling of the vacuum system and for removing air bubbles (see below). The components for the vacuum system are listed below.

− Constant vacuum pump (CVP, D3, Multi Channel Systems MCS GmbH, Reutlingen, Germany)
− Fine flow control (D2, Dosi-flow 10, P. J. Dahlhausen & Co. GmbH, Köln, Germany)
− Valves (v1, v2)
Valve v1 can either be a 2-way valve, or a 3-way valve (like the other valves) with one connector closed with a plug
− Tubing (inner diameter)
  ▪ 1×0.8 mm to connect v2–D1
  ▪ 3×∼1.6 mm to connect v2–D2, v2–v1, and v1–water bottle

− Connectors
− 1×18 ga blunt needle for 0.8 mm tubing at v2
− 1×plug for v1 if a 3-way valve is used
− Bottle with water

#### MEA Equipment

60-electrode perforated MEA with glass ring (Multi Channel Systems MCS GmbH, Reutlingen, Germany).

− MEA1060 system (Multi Channel Systems MCS GmbH, Reutlingen, Germany)

#### Specific equipment for in vitro electroretinogram (in vitro ERG) recordings

Visual stimulation only possible from below

− Ag/AgCl pellet reference electrode (Science Products E-201ML)
− Insulated connector (e.g. wire ferrule with shrink-on tube) and optical shield (shrink-on tube) for reference electrode
− Holder for reference electrode
− Pharmacology: 50 µM L-AP4 (Sigma A7929 or Abcam ab120002), 10 µM NBQX (disodium salt, Tocris 1044), 10 µM RS-CPP (Tocris 0173) to block synaptic transmission to bipolar cells, 100 µM BaCl_2_ (Sigma 342920) to block glial currents [15]

#### Other

Nitrocellulose filter papers (e.g. 13 mm diameter, 0.45 µm pore size, cat. no. HAWP01300, Merck Millipore, Billerica, USA).

## Experimental Procedure

### Step by step instructions

All specifications (e.g. flow control settings) are given for the equipment listed above and might have to be adjusted for different equipment. Although the procedure is explained for the 60-electrodes pMEA by Multi Channel Systems in combination with a MEA1060 amplifier, most steps could be transferred to experiments with other perforated MEA systems. For *in vitro* ERG recordings, most steps remain the same. Necessary adaptations and additional steps can be found in the section “Special considerations for *in vitro* electroretinogram recordings”.

Setting up for pMEA recordings (including retina preparation and hardware preparation) takes approximately 40–60 minutes depending on the complexity of the setup and the visual stimulation. Except for the steps involving the vacuum system and preparation of the filter paper, all steps are very similar to conventional MEA recordings. Further, no coating of the MEA with substances such as poly L-Lysin (used to fix the retina on non-perforated MEAs) is necessary for pMEA recordings. Overall, pMEA recordings require about 10 minutes more preparation time than conventional MEAs.

**IMPORTANT:** Whenever negative pressure is applied to the MEA chamber, make sure that this is either for only a very short time or that you are perfusing with fresh solution in parallel. Due to the shape of the MEA chamber and the surface tension of the solution, the solution level in the middle of the chamber – directly above the perforated membrane – is significantly lower than at the edges of the chamber (see inset in Fig. 2). Therefore, the MEA chamber always needs to be almost full; otherwise air will enter the vacuum system which can harm the retina and impede the constant negative pressure necessary for stable recordings.

#### Step 1: Filling of MEA chamber

**Figure 3:**
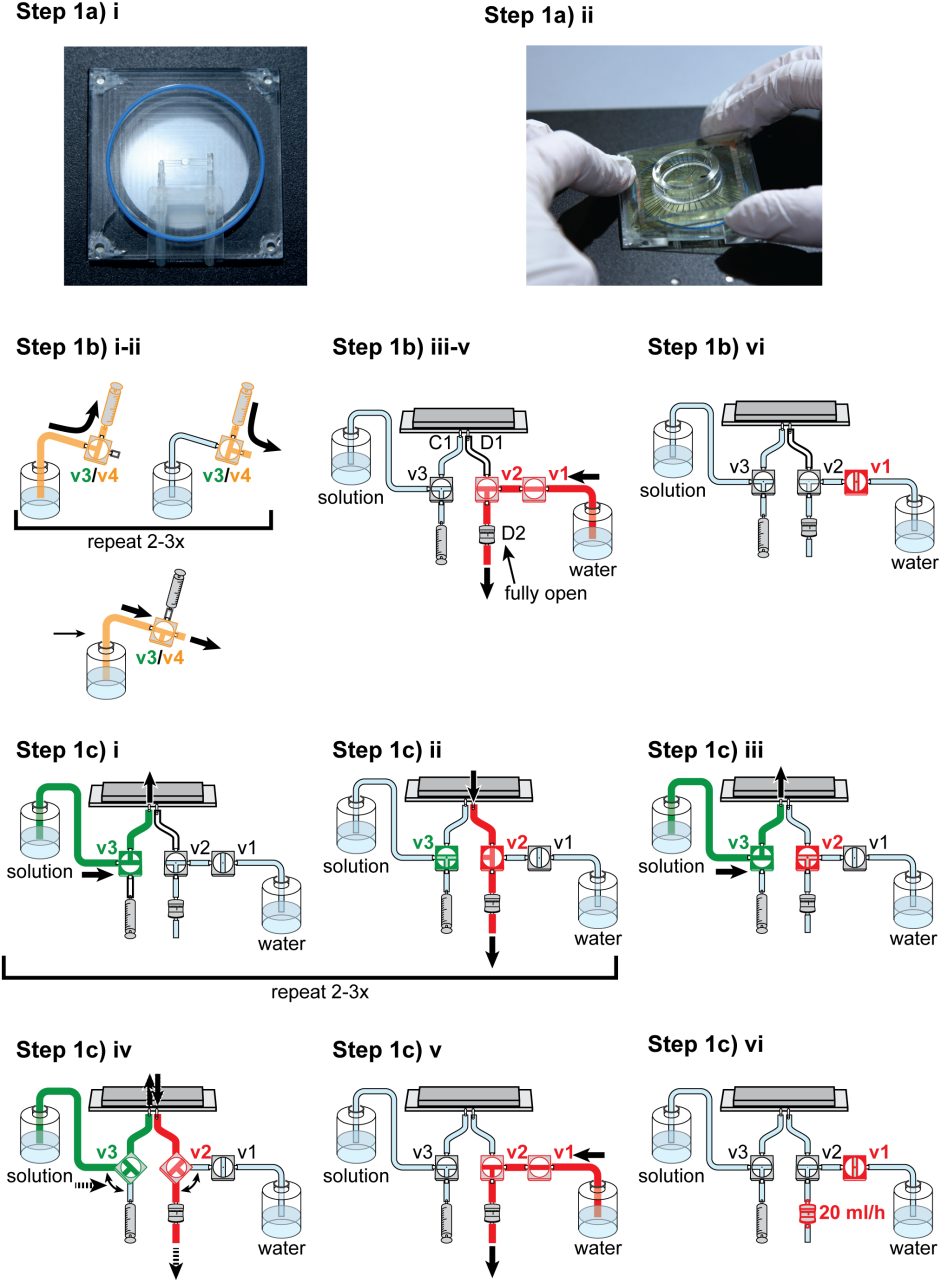
Experimental procedure Step 1: Filling of MEA chamber. **Step 1a)** Placing the MEA chamber on the baseplate. **Step** **1b)** Preparation of perfusion and vacuum. **Step** **1c)** Filling the MEA. Detailed description is given in the text.

**Illustrated in** **Figure 3** **.** In this step, the MEA amplifier is prepared, the pMEA is filled with solution, and the vacuum is established. Two aspects are crucial in this step: first, that the MEA baseplate is tightly sealed, and second, that all air bubbles are removed from the perfusion-vacuum system.

a. Place pMEA on MEA baseplate
  i. Place a rubber ring in the notch of the MEA baseplate.

**IMPORTANT:** Make sure that the ring is placed firmly in the notch

ii. Carefully place a clean and dry pMEA onto the ring. To do so, first place one edge of the MEA against the elevated edge of the MEA holder and then lower the MEA down onto the holder.

**NOTE:** By default, electrode number 15 is the reference electrode. Depending on the MEA amplifier this can be more or less easily changed. If you want to use the standard settings, make sure that the big reference electrode of the MEA is connected to recording pin 15 of the amplifier. This is achieved by placing the MEA with its reference electrode pointing to the right.

  iii. Carefully touch the MEA chamber and try to move it: it should not move if it is placed correctly, otherwise it might wobble on the rubber ring.
  iv. Close the amplifier.
b. Prepare perfusion and vacuum tubing
  - Upper perfusion: Wash and fill the tubing with physiological solution by the use of the syringe. Start gravity flow and then close the valve (v4). Do not yet connect it to the MEA chamber.
  - Lower perfusion: Wash and fill the tubing of the lower perfusion in the same way. Close the valve (v3) so that no solution flows into the MEA chamber.
  - Connect the lower perfusion to the left (shorter) cannula of the MEA baseplate and the vacuum tube to the right (longer) cannula.
  - Set the valves so that the connection of the vacuum system to the MEA is closed (v2) but the connection to the water bottle is open (v1). Open the fine flow control (D2) to maximal flow.
  - Place the free end of the tubing into a bottle with water and switch on the constant vacuum pump (set to ∼80–100 mbar). Remove major air bubbles by flicking at connections that might trap air.
  - When the tubing is filled with water, close the valve towards the water bottle (v1) so that all liquid flow is stopped.
c. Fill the MEA

In this step, the MEA and the cannulas of the MEA baseplate are washed, air bubbles are removed, and the MEA chamber is filled.

  i. Fill the MEA by opening the valve of the lower perfusion (gravity flow, v3).

**IMPORTANT**: Solution should enter the MEA chamber within approximately 1–2 seconds; otherwise the system is most probably not tightly sealed. If it does fill slowly, stop gravity flow immediately, and open the amplifier to prevent the electrode contacts (top plate) from getting wet. See also troubleshooting section 1.

ii. When the MEA chamber is almost full, close the valve of the perfusion (v3). Then open the valve of the vacuum system towards the MEA (v2) and thereby suck out the solution from the MEA chamber. Repeat filling and emptying 2–3 times to wash the MEA chamber.
iii. Fill the MEA chamber again.
iv. Repeatedly open and close the lower perfusion and the vacuum system (alternating) to remove air bubbles from the MEA chamber as well as from the cannulas and the tube connected to the vacuum cannula. Make sure that the MEA chamber does not run empty during this procedure (this will introduce new bubbles) and that it is filled almost completely after having removed all air bubbles. Close the valves to the baseplate (v2, v3).
v. Remove again air bubbles from the tubing by washing through with water (open v1) and “flicking off” air bubbles.

**IMPORTANT**: Make sure that ALL air bubbles are removed from MEA baseplate cannulas, the MEA chamber, and the vacuum system.

vi. Close all valves and set the fine flow control to approximately 20 ml/h.

**NOTE**: The setting of the fine flow control determines the negative pressure that will eventually be applied to the retina. The retina will tear and be sucked through the perforation if that pressure is too high.

#### Step 2: Retina preparation

Prepare the retina as usually for physiology experiments. Pay special attention to removing the vitreous thoroughly in order to get good electrode contact. Further, do not introduce any holes or tears into the retina during preparation, especially when removing the optic nerve. Also do not cut the retina since any incisions or holes in the tissue might cause turbulences in the liquid flow through the perforation or might counteract the establishment of the necessary negative pressure.

#### Step 3: Fixation on filter paper

**Figure 4:**
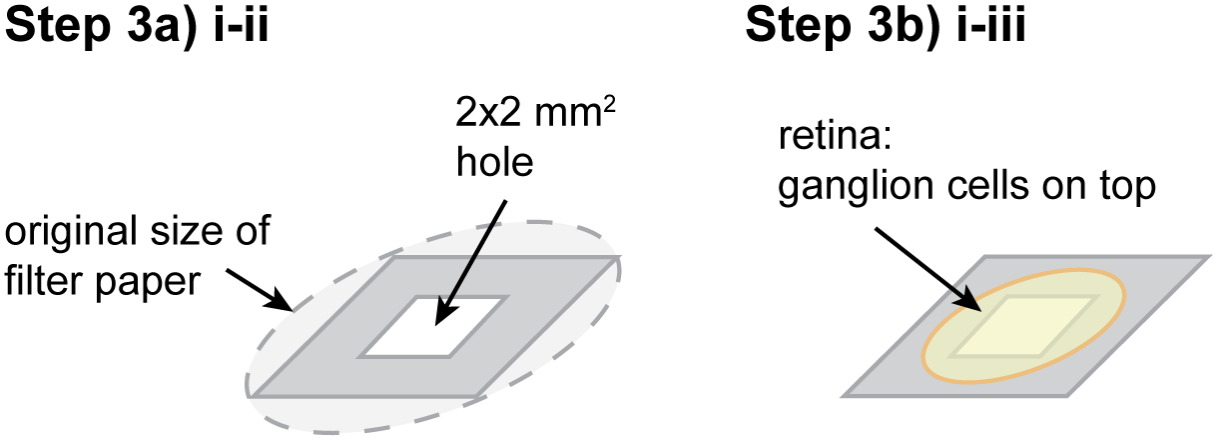
Experimental procedure Step 3: Fixation on filter paper. **Step 3a)** Preparation of filter paper. **Step 3b)** Fixation of retina on filter paper. Details are given in the text.

**NOTE:** Using a filter paper is essential for small retinas with a strong curvature, such as mouse retina. In the case of big retinas (e.g. rabbit, pig, cow, human), a filter paper is often not necessary. Here, the retina is cut into small pieces, which have almost no curvature and which can be placed directly on the electrodes by the use of brushes. Sometimes, even large retinas can roll up after having been cut into small pieces. In this case, a filter paper can be used to flatten the retina.

**Illustrated in** **Figure 4** **.** The filter paper is needed to flatten the retina without cutting the tissue.

- Prepare filter paper
  - Use a piece of a razor blade to cut a ∼2×2 mm hole into a filter paper.
  - Cut the edges of the filter paper.
- Place retina on filter paper
  - Center the retina with photoreceptors down over the hole in the filter paper.
  - Carefully press the edges of the retina onto the filter paper with forceps. Start in one corner, and then fix the opposite corner while carefully flattening the retina. You may hold down on the already fixed part with one pair of forceps while fixing the opposite side with a second pair.
  - Fix the rest of the retina while carefully flattening it.

#### Step 4: Transfer of retina to MEA chamber and setup

**Figure 5:**
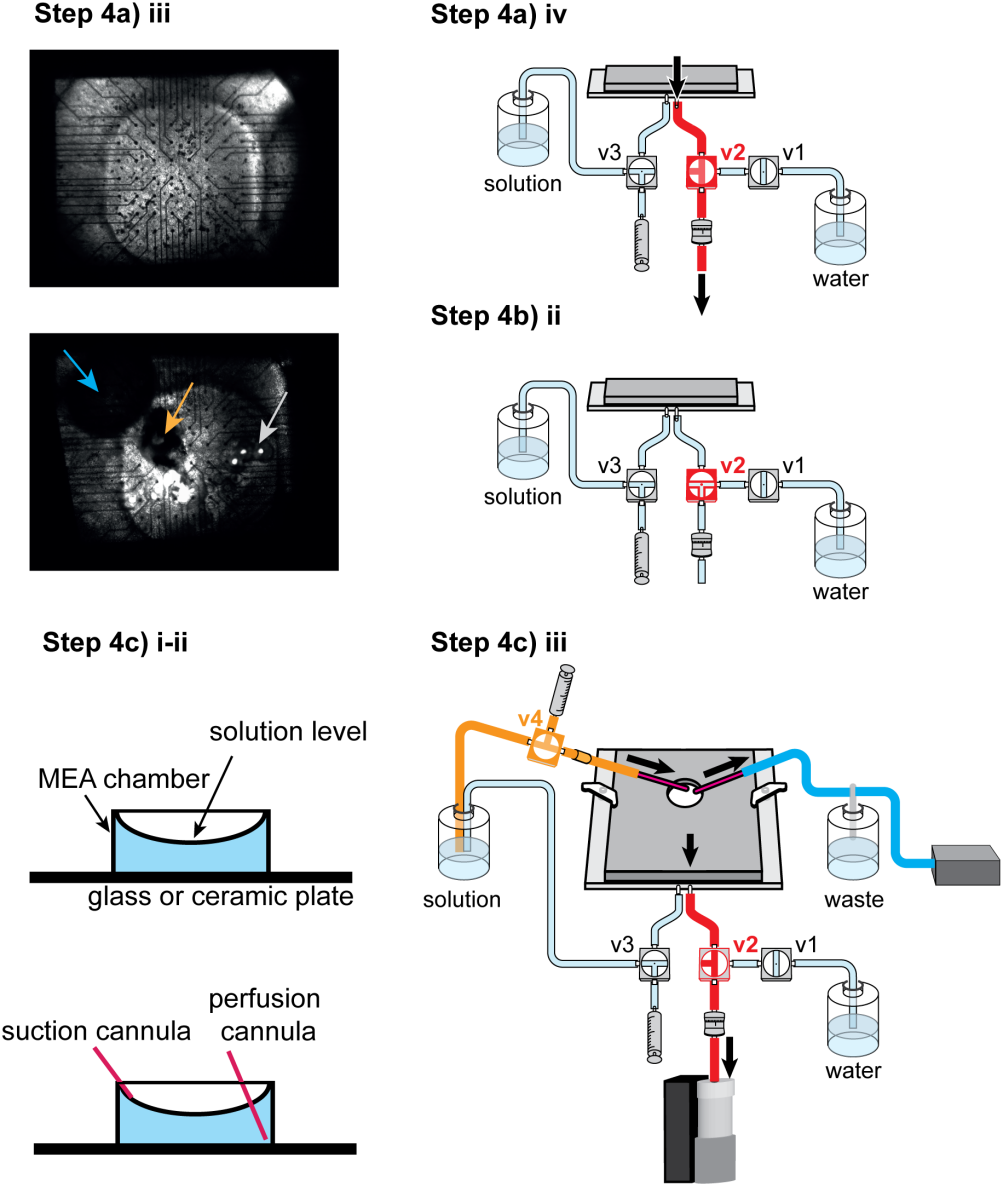
Experimental procedure Step 4: Transfer of retina to MEA chamber and setup. **Step 4a)** Placing the retina on the electrodes. **Step 4a) iii:** Top: Good MEA preparation. All electrodes are clearly visible; the retina looks homogeneous, flat, and without tears or holes. The retina and filter paper are nicely centered over the middle of the electrode array. Bottom: Bad MEA preparation with air bubble (blue arrow) and holes due to excessive negative pressure (gray arrow). Further, the filter paper is shifted towards the upper left corner. Orange arrow: optic nerve head. **Step 4b)** Transfer of MEA amplifier to setup. **Step 4c)** Installation of upper perfusion loop. Details are given in the text.

**Illustrated in** **Figure 5** **.**

a. Transfer retina to MEA chamber
  - Transfer the filter paper with the attached retina to the MEA chamber. This is best done with a spoon filled with solution so that the retina is always immersed in solution.
  - The filter paper should be oriented such that the ganglion cells are facing the electrodes. Usually, this means that the filter paper has to be turned upside down.
  - Center the retina over the electrodes. You can orient yourself using the layout of the wires connected to the electrodes.

**IMPORTANT**: Do not use forceps since you might destroy the electrodes or the perforated foil, instead use soft brushes to move the filter paper.

iv. Once the retina is centered, open the valve to the vacuum pump (v2). This will create negative pressure, pull the retina towards the electrodes, and hold it in place.

**IMPORTANT**: While applying negative pressure, the MEA chamber will slowly run dry. The next step has thus to be performed relatively swiftly.

b. Transfer MEA assembly to setup
  - If you performed the earlier steps outside of your recording setup, now move the MEA amplifier quickly into the setup and place it in the light path for visual stimulation.
  - Once the MEA is in place, close the valve to the vacuum pump (v2). The retina is now sticking to the perforated membrane and will not easily move. Nevertheless, you should avoid moving the MEA amplifier while no negative pressure is applied. The vacuum can stay switched off (i.e. valve v2 can stay closed) for the next steps to prevent the MEA chamber from running dry.
c. Installation of upper perfusion loop
  - Add the top perfusion cannula into the MEA chamber. Make sure it is on the bottom and at the edge of the chamber. Placing it on the bottom of the chamber prevents dripping of solution into the bath, which would cause turbulence and noise in the recordings. Placing it into the edge of the chamber helps to prevent touching and damaging the retina.
  - Add the suction cannula so that its opening is at the desired solution level (as high as possible without risking overflow).
  - Switch on the top perfusion (v4) and the suction, and open the valve to the vacuum pump (v2).

**NOTE**: The lower perfusion is not used during the experiment. Flow through the perforation would cause turbulences and hence noise.

**IMPORTANT**: The flow speed of the upper perfusion has to be at least as fast as the suction speed of the (lower) vacuum pump, otherwise the MEA chamber will run dry. However, it is advisable to have the upper perfusion at a higher speed. The solution level in the MEA chamber will then rise up to the level at which solution is sucked away by the upper suction. Therefore, the cannula of the upper suction has to be placed low enough to prevent overflow on one hand, and high enough to ensure sufficient liquid level.

#### Step 5: Check electrode contact

**Illustrated in** **Figure 5** **and** **6** **.** To check the contact of the retina with the electrodes, one can use visual inspection and check the signal-to-noise ratio.

**Figure 6:**
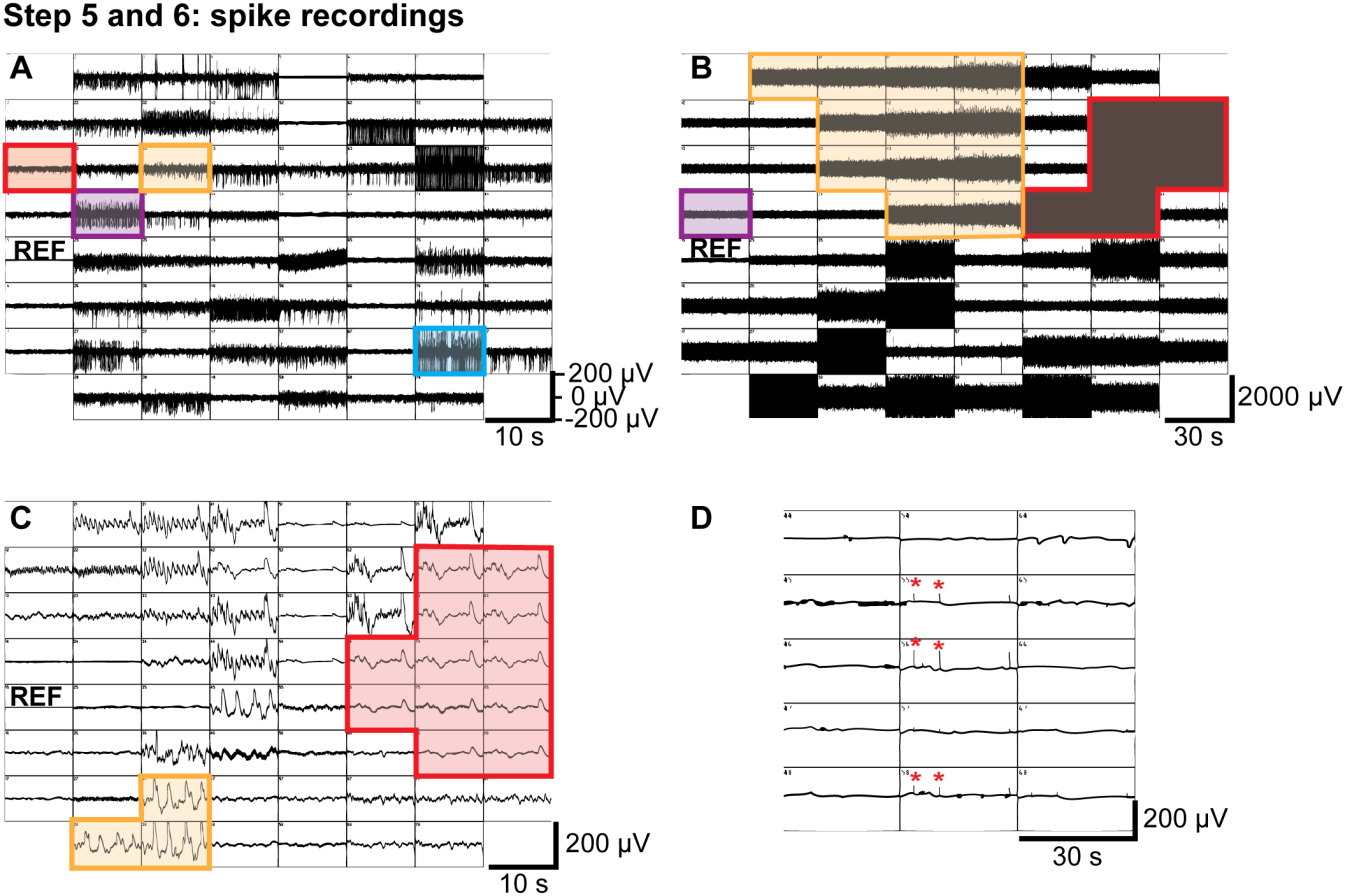
Experimental procedure Steps 5 and 6: Recording data (Spike recordings). **A)** Snapshot of a 500 Hz high-pass filtered MC_Rack display. Spiking activity with good signal-to-noise is visible on many electrodes. **B)** Snapshot of MC_Rack display after overflow. Noise with amplitudes of 200 to over 1000 µV due to wet electronics is visible on most electrodes. **C)** Snapshot of MC_Rack display several hours after strong overflow. Slow noise on many electrodes is visible either if the electronics is not fully dry yet or when the electronics has been irreversibly harmed. **D)** Snapshot with slow fluctuations and spike-like noise peaks (red asterisks). See text (Step 5 and 6, troubleshooting) for details.

- Visual inspection

If the retina can be imaged in your setup (e.g., with an infrared camera system), visual inspection of the retina can be used to judge preparation and contact of retina with electrodes (photographs in Fig. 5). Contact with electrodes is usually good if the retina looks flat and if most or all electrodes can be seen through the retina. However, in the region of the optic nerve, the retina is often not totally flat. Now also the visual stimulus can be focused on the photoreceptors and centered on the middle of the electrode field.

b. Setting up MC_Rack software

Consult manuals provided by Multi Channel Systems for installation and setup of MC_Rack for recording of ganglion cell activity. In general, it is advisable to have a Longterm Data Display showing unfiltered activity for each electrode. In addition, it is useful to have a Data Display showing high-pass filtered data, i.e. spiking activity. To implement this, add a filter before the display with a 500 Hz high-pass Butterworth 2^nd^ order filter. See also step 6 (spike recordings) and “Special considerations for *in vitro* electroretinogram recordings”.

c. Signal-to-noise ratio

In addition to the *number* of electrodes with activity, the *amplitude* of this activity is crucial for the success of subsequent spike sorting. If the retina is flattened well, all electrodes should show activity (exceptions: those lying directly under the optic nerve, and the ground electrode). When inspecting the high-pass filtered data, the noise level should not exceed 20 µV and spiking activity should have an amplitude of 100–250 µV (signal-to-noise ratio of at least 5; see Fig. 6A). As a rule of thumb, the signal is strong if spiking activity is well visible or even filling the display window when the display y-axis is set to 200 µV; the spikes should be sortable for amplitudes of at least 100 µV. Raw data with smaller activity will most probably not be sortable.

**NOTE:** Spontaneous activity of ganglion cells can be very sparse in the beginning of the experiment. The retina should always be allowed to settle and adapt to the new environment (negative pressure, change in temperature, …) for at least 20 minutes before recording data. Usually, spontaneous activity appears during this time if it has not been present from the beginning. If there are very few spikes, the retina can be probed with some light stimuli and the elicited spikes can be used to check signal-to-noise ratios. If activity is still sparse and/or signal-to-noise ratio is low, increase the negative pressure slightly by changing the flow control to 30–50 ml/h. Also consult the troubleshooting section for possible counter-measures.

#### Step 6: Recording data

**Illustrated in** **Figure 6** **. Spike recordings:** In most cases, one uses MEAs to record spiking activity from ganglion cells. As mentioned above, when using the MC_Rack software, it is useful to show the data in two displays while recording: (1) Longterm Display with unfiltered data. Set the display y-axis to 500 µV for good overview. (2) Data Display with high-pass filtered data for better visualization of spiking activity. Add a 2^nd^ order Butterworth 500 Hz high-pass filter before a Data Display and set the y-axis to 100 or 200 µV. Figure 6A shows such filtered electrodes with the y-axis set to 200 µV. In optimal recordings, all electrodes would have activity with amplitudes such as the electrode marked in “blue”. A signal-to-noise ratio and activity level like on the “purple” electrode is also sufficient for good spike sorting. Whether the spikes on the “orange” electrode are sortable will depend on how distinguishable the waveforms of various cells and of the noise are in each particular case. On the “red” electrode, the signal-to-noise ratio is clearly too small. The reference electrode 15 is on the left.

**NOTE:** Usually, the MEA is placed in the setup such that the vacuum and perfusion cannulas are at the “front” (i.e., facing the researcher). Note that in this configuration the physical reference electrode 15 is on the right side of the MEA chamber and electrodes 15–18, 25–28, 35–38 etc. will be in the upper half of the MEA (60-electrode pMEA, 8×8 layout). The orientation of electrodes in the MC_Rack displays is mirrored compared to that: the reference electrode 15 is on the left, electrodes 11–14, 21–24 etc. are displayed in the top half. Consequently, when showing a stimulus which moves from the top left to the bottom right corner of the MEA chamber, the retinal activity will move from the bottom right to the top left corner of the MC_Rack display.

#### Step 7: Removing the retina

The retina is a relatively thin tissue. It can thus rarely be removed entirely from the pMEA after the recording. In general, removing the retina works best when the vacuum system is off, the lower perfusion is switched on and the flow is slightly increased via the syringe. Use a very fine brush to help removing the retina from the recording chamber. Subsequent analysis of the tissue (e.g. histological stainings) is only possible if the negative pressure is kept as low as possible during the experiment and if the retina is removed very carefully from the perforated foil. This is easier for thicker (healthy) and bigger (species-dependent) retinas; however, we also performed experiments with very thin and vulnerable degenerated retinas (rd1 mouse model with quickly degenerating rods and cones). Even these retinas could be removed and stained after the recordings when only little negative pressure had been applied during the experiment (data not shown).

### Special considerations for *in vitro* electroretinogram (ERG) recordings

Electroretinography (ERG) is the most common electrophysiological technique for recording retinal activity in both human patients and living animals. ERG signals reflect mainly the activity of cells oriented vertically in the retina, namely photoreceptors, bipolar cells and Mueller glia. The pMEA system can be configured to record an *in vitro* electroretinogram. For this, an additional reference electrode is added to achieve a recording configuration in which the retina is “sandwiched” between recording electrode(s) and reference electrode to record transretinal potential changes. Follow all procedures as outlined above for spike recordings and add the following steps:

#### Step 1a) iii. Place pMEA on MEA baseplate

**Figure 7:**
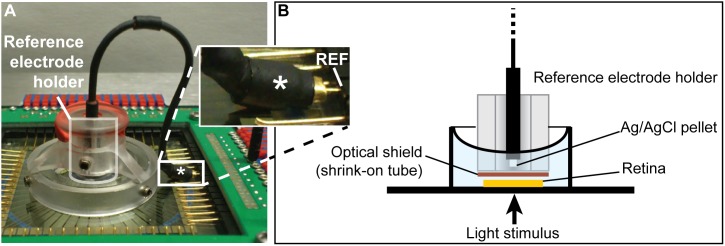
Additional steps for *in vitro* ERG recordings. **A) Additions to Step 1:** The AgCl reference is positioned over the MEA by a reference electrode holder and is attached to pin 15 (REF) by a wire ferrule insulated by shrink-on tubing (asterisk). **B) Additions to Step 5:** Schematic of the reference electrode and its holder as shown in A. Note the optical shield needed to avoid photoelectric artifacts resulting from light hitting the reference electrode.

**Illustrated in** **Figure 7** **.** An external reference electrode has to be attached to recording pin 15 of the amplifier before the next step. A wire ferrule soldered to an Ag/AgCl reference can be used to connect to pin 15. Shrink-on tube around the wire ferrule insulates from the MEA chamber’s internal reference contact.

#### Step 5a) Visual inspection

After the stimulus is centered, the external reference electrode has to be put into the MEA chamber. Placing the reference electrode before this step would obscure the camera’s view and make stimulus centering impossible (in configurations like in an upright microscope). It might be necessary to once again remove the upper perfusion/suction to place the reference electrode and reposition it after the external reference is in place. The Ag/AgCl pellet of the reference has to be positioned 2 to 3 mm above the center of the MEA electrode field and optically shielded from direct stimulus illumination to prevent photoelectric artifacts in the reference electrode. The upper suction has to be adjusted such that the solution level is high enough to completely immerse the Ag/AgCl pellet of the reference in the solution. Perforations in the optical shield that allow solution to pass but do not compromise the optical shielding, can help to achieve this. The suction has to be carefully adjusted so the solution level does not fluctuate; otherwise there will be periodic low frequency noise that can spoil the *in vitro* ERG data (see troubleshooting section).

#### Step 6) Recording data

**Figure 8:**
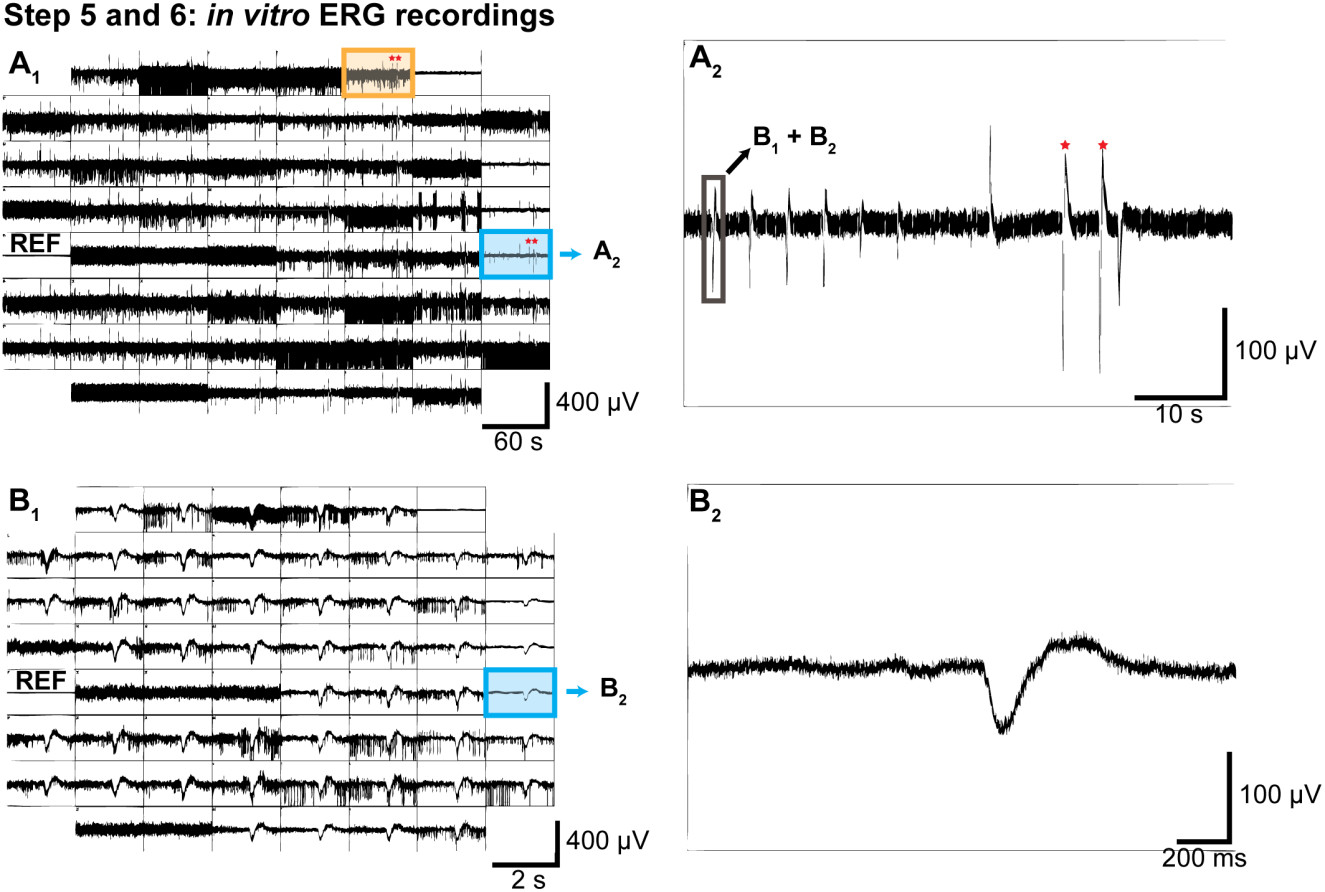
Experimental procedure Steps 5 and 6: Recording data (*in vitro* ERG recordings). **A_1_)** Snapshot of the Longterm Data Display (raw data) from MC_Rack. Note that on most electrodes the ganglion cell spikes mask the *in vitro* ERG responses (e.g. the electrode marked in orange). Only the highest contrast flash elicits a response that is visible on most electrodes (red asterisks), while on some electrodes without ganglion cell spikes the *in vitro* ERG responses are clearly visible (electrode marked in blue). Reference electrode 15 (REF) is on the left. **A_2_)** Zoomed view of the electrode marked in blue from panel A_1_ showing the responses to flash stimuli of different contrast (highest two contrasts marked with red asterisks). The low-pass filtered data around the time highlighted by the box is shown in B_1_+B_2_. **B_1_)** Data Display with 200 Hz low-pass filter applied. There is a clear response on almost all electrodes. Not all spikes get filtered out by the low-pass filter. Note the different time scale than in A_1_. **B_2_)** Zoomed view of the electrode marked in blue from panel B_1_ that shows a very clear low frequency *in vitro* ERG response without contamination by ganglion cell spikes.

**Illustrated in** **Figure 8** **.** For *in vitro* ERG recordings, the Data Displays in MC Rack are set up in a similar way as described above, except that the filter setting for the second Data Display is set to low-pass filter. This eliminates some of the ganglion cell spiking responses for clearer visualization of the slow *in vitro* ERG responses. Add a 2^nd^ order Butterworth 300 Hz low-pass filter before the Data Display and set the y-axis to 100 or 200 µV.

In our experiments, synaptic transmission to bipolar cells and glial currents were pharmacologically blocked to isolate the field potentials generated by photoreceptor activity. Figure 8 shows example responses to several flashes with different contrasts (panels A) and a close-up view of a single flash response (panels B) from a good *in vitro* ERG recording.

### Troubleshooting

Due to the two perfusion loops, solution leakage or overflow is encountered more often than with standard MEAs. Thus, most issues encountered during pMEA recordings will be linked to electronics which got in contact with solution, and will be recognizable in the noise level of the electrodes. In this troubleshooting section we discuss the 10 most frequent problems. The titles indicate the main aspect which will be noticed during MEA recordings. Each issue is then followed by a description of its possible causes, the detailed symptoms which can be observed, and the required actions.

#### 1. MEA chamber fills very slowly during Step 1c) i

**Possible cause (1):** Leakage due to insufficient seal between MEA chamber and the baseplate (Step 1a) i). The solution from the lower perfusion can fill the space between the MEA chamber and the MEA baseplate, rather than being pushed quickly through the perforation.

**Detailed symptoms (1):** A long delay is observed between opening the lower perfusion and filling of MEA chamber.

**Required actions (1):** Immediately stop lower perfusion! Open the MEA amplifier immediately in order to prevent the solution from reaching the recording pins of the amplifier. MEA baseplate and the rubber ring should be dried completely and the MEA chamber should be placed again such that it does not move. Minor leakages are hard to detect while filling the MEA chamber and will reach the recording pins only later during the recording. These slow leakages are, however, very rare.

**Possible cause (2):** Mishandling of the MEA chamber (e.g. applying a relatively large force) can weaken the seal between MEA ring (forming the wall of the chamber) and MEA glass plate. This can introduce local gaps in the glue between wall and floor of the MEA chamber from where the solution can leak.

**Detailed symptoms (2):** A high latency is observed between opening the lower perfusion and filling of MEA chamber. Solution usually leaks from a specific region where the seal is weak.

**Required actions (2):** Immediately stop lower perfusion! Open the MEA amplifier immediately to avoid solution reaching the recording pins. Experiment cannot be continued with this MEA chamber which should be sent to Multi Channel Systems for maintenance.

**NOTE:** Often the leak is not detected while filling the MEA but is reflected later in the signal as noise on a group of electrodes.

#### 2. Noise observed on (almost) all electrodes

**Possible cause:** Overflow or leakage due to badly adjusted upper perfusion, negative pressure and suction. In this case the MEA chamber can either run dry, thereby damaging the tissue and introducing turbulences, or it can overflow and solution can reach the recording pins.

**Detailed symptoms:** Overflow or leakage lead to high amplitudes of noise in most of the recording electrodes, specifically the recording pins that are in contact with the solution. Figure 6B shows such a case. As visible in these traces, some electrodes are affected so strong (marked in red) that the noise is filling the whole display even when setting the y-axis to 1000 µV. But even on the electrode marked in purple the noise level is much higher than usual with amplitudes of around±200 µV. Often, distinct groups of electrodes have similar noise patterns (here one group in red and one in orange). This can be caused by “local” leakage/overflow when only some of the pins have become wet. Alternatively, even when all pins are wet, the solution might seep into the electronic housing with different speeds and might thus affect the electronics of different channels with different delay.

**Required actions:** In the case of overflow, the recording should be stopped and the MEA amplifier should be removed immediately. If overflow was detected as soon as it started, the recording pins should be dried and carefully cleaned with a wet cotton swab (deionized water and/or alcohol). A tissue paper can be used to suck out solution from the small openings where the recording pins are connected to the MEA amplifier. If the overflow was detected at a later stage, a relatively large amount of solution could have entered the MEA amplifier. The whole amplifier should be placed in deionized water for several hours to wash out the salts, after which it requires 1–2 days to dry. The amplifier should then be tested using the model probe supplied with the amplifier. If the signal from the amplifier appears noise free (less than 20 µV amplitude) and does not show any slow fluctuations, it should be tested with a MEA chamber (filled with PBS or other physiological solution). If the electronics are not completely dry, localized slow noise waves, again affecting subgroups of electrodes (Fig. 6C: one group in red, one in orange), can be detected. However, if this noise persists after 2–3 days, most probably not all salts were washed out which possibly harmed the electronics. In such case, the MEA amplifier needs to be submitted to Multi Channel Systems for maintenance.

#### 3. Stable high frequency noise on one or several electrodes

**Possible cause:** One or several electrodes are deteriorated either due to frequent and/or long-term use. Alternatively, they can also be harmed by use of forceps during placing or removing the retina.

**Detailed symptoms:** In contrast to noise caused by overflow, deteriorated electrodes often show very stable high frequency noise. Even if only one electrode is affected, the noise might spread to neighboring electrodes.

**Required action:** Refer to manuals provided by Multi Channel Systems for hardware or software based grounding of the affected electrodes.

#### 4. Fluctuations/noise on a group of electrodes

**Possible cause (1):** Air bubbles under the retina, either above or below the perforated foil, can lead to significant noise levels. These bubbles usually arise either when air is trapped in the perfusion tubing or when the solution level in the MEA chamber becomes too low.

**Detailed symptoms (1):** Due to the continuous negative pressure, such air bubbles – once they are trapped in the MEA chamber – move around, change in size, and might disappear and reform constantly. They can easily be recognized when imaging the retina in the MEA chamber (Fig. 5, Step 4a) iii). These bubbles can often induce big voltage fluctuations on several electrodes, can cause large noise amplitudes or inhibit contact between solution/tissue and electrodes (electrode traces are flat, as if connected without solution and retina).

**Required Action (1):** If air bubbles are caused by too little solution in the MEA chamber, the chamber should be filled immediately by increasing the flow speed of the upper perfusion and/or moving the suction cannula further up. If the bubbles do not disappear, the following two counter-measures can be applied:

- *Increasing the negative pressure (short term)*

Increasing the negative pressure might “suck out” the air bubbles through the perforation. Make sure that your perfusion is fast enough so that the solution level does not drop again. Watch the retina closely to not increase the negative pressure too much, which might tear or destroy the retina. Try switching back to lower negative pressure once the air bubbles are removed.

ii. *Opening the lower perfusion*

Opening the lower perfusion can push out air bubbles from the space between retina and electrodes into the MEA chamber. This is often more effective when no negative pressure is applied; however, care should be taken not to wash away the retina.

Parallel application or quickly alternating the above mentioned measures can sometimes facilitate removal of the bubbles. It is advisable to image the retina and to observe noise levels and activity on the electrodes during this process.

**NOTE:** Air bubbles often cannot be removed and the experiment has to be stopped. The described measures are only advisable before recording data since the turbulences caused by the air bubbles as well as by the counter-measures will move the retina and might change the footprint of the recorded cells on the MEA electrodes.

**Possible cause (2):** Starting overflow or leakage due to incoherent upper perfusion, negative pressure and suction.

**Detailed symptoms (2):** As the overflow/leakage starts, only a group of electrodes is affected. In contrast to deteriorated electrodes, the noise is often a mixture of low and high frequencies and might show large fluctuations.

**Required actions (2):** Immediately stop the experiment and open the MEA amplifier. Check troubleshooting point 2 for further procedures.

#### 5. High baseline noise on all electrodes

**Possible cause:** Poor grounding of the upper perfusion or suction system.

**Detailed symptoms:** Noise levels above 20 µV on all electrodes. Usually without big fluctuations.

**Required actions:** Refer to manuals provided by Multi Channel Systems for improving grounding.

#### 6. Synchronous spike-like activity on all or a group of electrodes

**Possible cause:** Poor grounding of the upper perfusion can lead to spike-like activity (see Fig. 6D).

**Detailed symptoms:** Synchronous, regular, and sparse high frequency noise is observed on a group or all electrodes e.g. due to regular dripping of solution from the perfusion system.

**Required actions:** Check manuals provided by Multi Channel Systems for improving grounding.

#### 7. Low signal-to-noise ratio

**Possible cause:** Poor retina preparation and placement or insufficient negative pressure can often lead to low signal-to-noise ratio.

**Detailed symptoms:** Spiking activity is visible, but very small.

**Required actions:** Once the retina is placed in the MEA chamber, contact can only be improved by increasing negative pressure. The flow control should not be set to values higher than 40–60 ml/h (depending on the species and retina condition). However, short term application of higher pressure (up to 100 ml/h flow) might increase signal-to-noise ratio. The negative pressure should not be changed during the recordings since it can move the retina and change the footprint of the recorded ganglion cells.

**NOTE:** During retina preparation, the vitreous should be completely removed from the retina and the retina should be properly flattened and carefully fixed on the filter paper.

#### 8. Retina is suddenly out of focus (when imaging from top)

**Possible cause:** The solution level is rising due to too fast upper perfusion or impaired suction.

**Detailed symptoms:** In the beginning of the experiment, while letting the retina settle down, the retina appears suddenly out of focus when imaged from top (sudden blurring of the camera image).

**Required actions:** Immediate adjustment of upper perfusion, suction, and/or negative pressure prevents overflow in this case. Noise levels have to be observed carefully to ensure that the solution does not reach the recording pins.

**NOTE:** The described procedures refer to very sudden blurring in the first 10–20 minutes after switching on the perfusion system. After many hours of recording, the solution level might have changed slightly so that the retina appears out of focus. In this case, usually no counter-measure is required.

#### 9. Low frequency noise on some or all electrodes 1–2 days after overflow

**Possible cause:** Following an overflow, the electronics in the amplifier needs 1–2 days to dry completely. Low frequency noise indicates that either the electronics is not yet completely dry or that it has been harmed from salts.

**Detailed symptoms:** Slow noise waves, often affecting subgroups, are visible on most or all electrodes (Fig. 6C: one group in red, one in orange).

**Required actions:** The amplifier should be left to dry for an additional day. However, the noise can still persist after 2–3 days if not all salts were washed out which possibly harmed the recording pins or the electronics. In such case, the MEA amplifier needs to be submitted to Multi Channel Systems for maintenance.

#### 10. Noise during ERG recordings

**Possible cause:** The external reference electrode is very sensitive to fluid level changes in the MEA chamber. Periodic fluctuations of the fluid level can be caused by use of a peristaltic pump for perfusion or, more importantly, by intermittent interruptions in the suction stream. This is usually caused by periods of rapid suction of solution until the fluid level drops below the suction cannula opening, followed by no solution being sucked out until the fluid level gets high again.

**Detailed symptoms:** Simultaneous high amplitude signals on most electrodes that often appear in regular intervals of up to tens of seconds. The noise signals can resemble ERG responses or look like spikes but can also have less stereotypical shape. Sometimes the noise signals look similar to sinusoidal 50 Hz noise.

**Required actions:** Adjust the depth and angle of the suction cannula. Ideally, an uninterrupted suction stream should be achieved that sets the fluid level in the MEA chamber such that the external reference electrode is fully immersed in solution at all times. This might require several adjusting steps and longer waiting times until the solution level stabilizes, and changes to the suction cannula might be necessary.

## Anticipated results

### pMEAs provide good signal-to-noise ratios

The vacuum applied through the perforation of pMEAs greatly enhances the contact between the tissue and the electrodes. In our experience, on good recording electrodes, we can detect and properly spike-sort one to three cells per electrode. On some electrodes, no spikes might be visible because blood vessels or the optic nerve lie on these electrodes. Our pMEAs have 59 recording electrodes. After multiple experiments, some electrodes might deteriorate and might not be usable anymore due to increased electrical noise. Good signal-to-noise ratios are crucial for most spike sorting algorithms since they usually depend on amplitude and principal component analysis of the recorded spikes. To get an estimate of the number of recorded cells that one might expect in such experiments, we counted the number of extractable cells in 153 recordings from mouse retina (without pre-selecting “good” and “bad” experiments), and found on average 38±18 cells (median ± standard deviation) with 6 sorted cells in the worst and 110 cells in the best case. Pig (domestic and minipig) and human retina recordings often had even better signal-to-noise ratios and therefore lead to more sortable cells. In pig retina, we found on average 48±31 cells (range: 13–109, n = 20 retinal pieces), and in human retina 51±32 cells (range: 6–154, n = 35 retinal pieces).

### pMEAs allow stable long-term recordings

**Figure 9:**
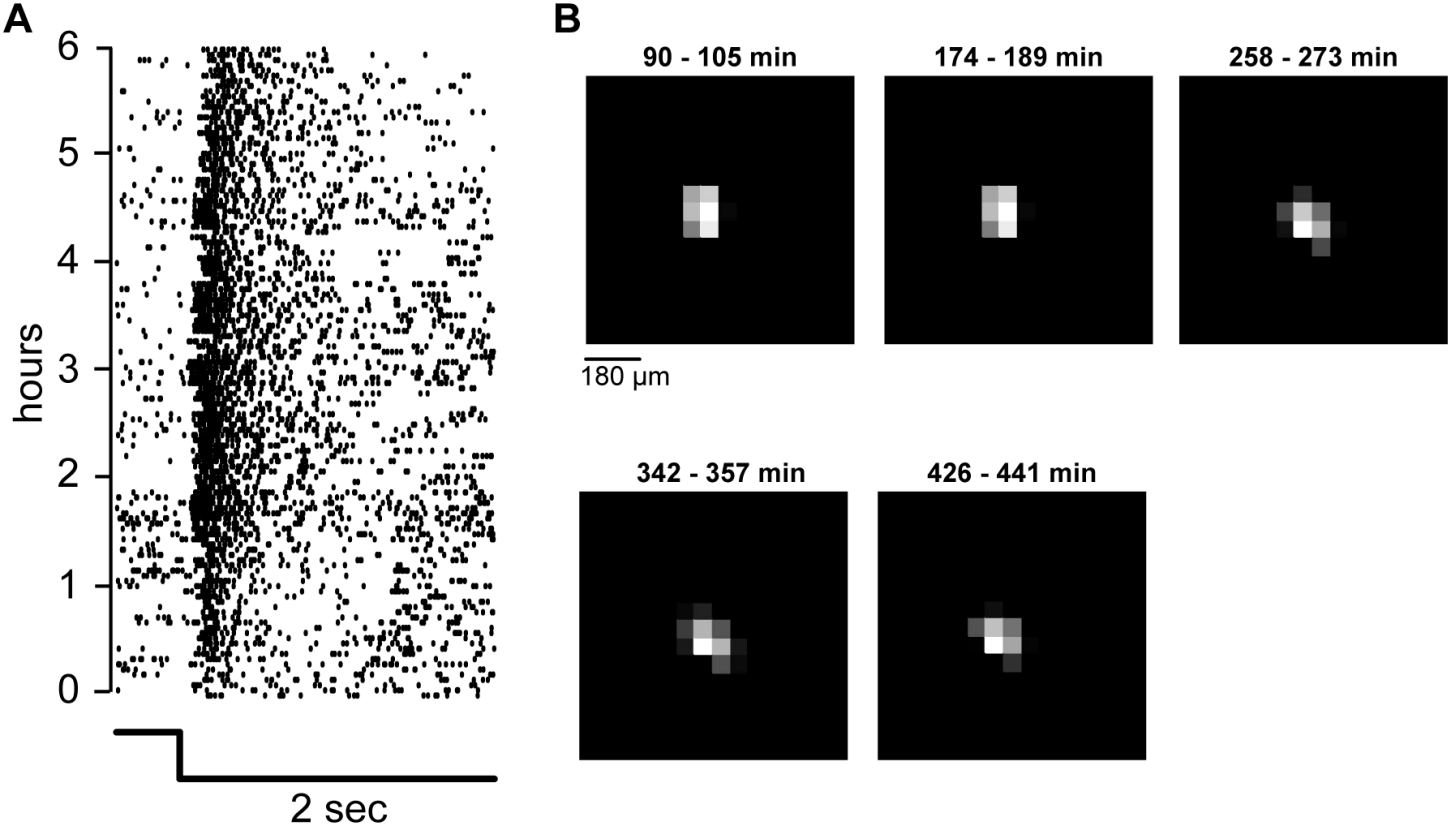
Recording stability. **A)** Responses of one ganglion cell to a step in contrast over 6 hours. A two second light decrement step has been shown >120 times over a period of 6 hours. Each dot in the raster plot represents one spike produced by the ganglion cell. The ganglion cell stably responded to the stimulus during the whole recording time. Changes in latency and number of spikes are due to different mean brightness levels used during the experiment. **B)** Receptive field of one ganglion cell calculated from checkerboard stimuli. 15×15 checkers out of 40×40 shown here. The stimulus has been repeated approximately every 90 minutes. Time above each receptive field map: presentation time of the checkerboard stimulus (0 min = beginning of experiment). The receptive field location and shape was stable during the whole 8 hours, indicating that the retina did not move significantly.

Nutrient and oxygen supply is crucial for the survival of ganglion cells. If ganglion cells do not receive enough oxygen and nutrients, their responsiveness might change and/or decrease over time which leads to instability of light responses in long-term recordings. During conventional MEA experiments, the supply to the ganglion cell might be insufficient. It has been shown by Egert et al. that with pMEAs, the oxygenation of the bottom layer cells is greatly enhanced, and it can be assumed that the same is true for nutrient supply to these cells [8]. We additionally show the viability of the ganglion cells by example data from a long-term recording. We showed various light stimuli to a mouse retina on a pMEA during 6 hours and recorded ganglion cell responses. A very simple stimulus – namely a full-field step in contrast – was part of the stimulus set and has been presented over 120 times during these 6 hours. Fig. 9A shows the responses of one ganglion cell to all these repetitions. As visible in the raster plot (every dot represents one spike), the cell responded to every repetition of the stimulus, also after 6 hours of continuous recording. The differences in response latencies are due to switches in absolute brightness which have been part of the stimulus protocol.

### pMEAs prevent movement of retina

The third advantage of applying negative pressure to the retina is that movement of the tissue is prevented. We recorded ganglion cell responses to binary checkerboard stimuli to calculate receptive fields and to visualize tissue movement. The checkerboard stimulus consisted of 40×40 checkers with 60 µm edge length. Fig. 9B shows the spatial receptive field of a single ganglion cell, repeatedly calculated from 15 min of checkerboard stimulus, presented every 90 minutes during this 8 hour recording. Location and shape of the calculated receptive fields are very stable. Note that slight changes in shape are also due to different absolute brightness levels used at each presentation (from scotopic to photopic).

## Conclusions

In this article we provide a step-by-step procedure for retina recordings with perforated MEAs. Although the preparation and adjustment of the additionally required perfusion and vacuum system might seem complicated at a first glance, the additional time required for perforated compared to conventional MEA recordings amounts to only around 10 minutes. Further, little additional material is needed when switching from standard to perforated MEA recordings. Finally, pMEAs provide better oxygenation of ganglion cells which allows for long-term recordings, and the applied negative pressure facilitates flattening and placement of small retinas with strong curvature. Especially when isolating single cell responses from MEA recordings, the user will appreciate the resulting high signal-to-noise ratio in pMEA recordings.
